# Characterizing local and systemic exposure to clobetasol propionate in healthy subjects and patients with atopic dermatitis

**DOI:** 10.1002/bcp.70102

**Published:** 2025-07-11

**Authors:** Janna K. Duong, Sven van Dijkman, Gary Ong, Alexandra Marta, Adriana Ceci, Ernesto Bonifazi, Oscar Della Pasqua

**Affiliations:** ^1^ Clinical Pharmacology Modelling & Simulation GSK Australia; ^2^ Clinical Pharmacology Modelling & Simulation GSK UK; ^3^ Pharma Research and Development Global Medical, GSK Singapore; ^4^ Pharmacological Research Foundation ‘Gianni Benzi’ Italy; ^5^ Past professor of Pediatric Dermatology “Aldo Moro” University Bari Italy; ^6^ Clinical Pharmacology & Therapeutics Group University College London London UK

**Keywords:** atopic dermatitis, clobetasol propionate, corticosteroids, PBPK modelling, systemic exposure, tissue exposure

## Abstract

**Aims:**

There is a potential risk of systemic side‐effects with the use of potent corticosteroids, such as clobetasol propionate (CP). This concern is of particular interest in paediatric patients. The aim of this study was to develop and verify a physiologically based pharmacokinetic (PBPK) model to describe the local and systemic exposure to CP following topical application over a period of up to 4 weeks.

**Methods:**

Data from 12 clinical studies in healthy adult subjects and patients with atopic dermatitis (AD) were available for this investigation. A PBPK model including skin barrier impairment was developed to predict the effect of AD lesions on systemic exposure. Simulation scenarios were then evaluated to assess the effect of formulation, skin condition and surface area (5%–60% of body surface area [BSA]) on systemic exposure.

**Results:**

The PBPK model described the absorption and disposition characteristics of CP. Mean clearance, volume of distribution (V_ss_) and renal clearance were 27 L/h, 2.34 L/kg and 0.12 L/h, respectively. The half‐life of CP after topical application was significantly longer than after an IV dose (20.8 *vs*. 5.2 h). Systemic CP concentrations were higher with increasing surface area and skin barrier impairment. However, CP accumulates in the stratum corneum as the skin barrier function improves during treatment.

**Conclusions:**

Systemic and local exposure to CP increases with impaired skin barrier in AD and larger application area. Given the recommended maximum dose of 50 g per week, CP should not be applied to an area of more than 30% of the BSA. Availability of this model will allow extrapolation of CP pharmacokinetics from adults to children.

**What is already known about this subject**

- Clobetasol propionate (CP) is a potent topical corticosteroid (TCS) indicated for the treatment of atopic dermatitis (AD) in adults and children.
- Current recommendations on the use of TCS is based on finger‐tip units (FTU) and the maximum recommended dose for CP cream or ointment (0.05% w/w) is 50 g per week with a maximum treatment duration of 2–4 weeks.
- If used at high doses and over a prolonged period, high systemic concentrations of CP can cause side‐effects such as hypothalamic–pituitary axis (HPA) suppression and Cushing's syndrome.
- The local and systemic pharmacokinetics, as well as the implications of skin flares on the relative bioavailability of CP are unknown following clinical doses.

**What this study adds**

- This dermal PBPK model describes in a mechanistic manner both the local and systemic exposure of CP following application of two topical formulations in adult subjects.
- It appears that changes to skin barrier due to lesional skin increase the absorption of CP by 3.7‐fold relative to normal skin, resulting in higher plasma exposure.
- CP should only be applied to a maximum application area of 30% of BSA daily for 2 weeks as exceeding this would exceed the maximum recommended dose of CP (50 g weekly).
- The final model parameterization in adults offers an opportunity to evaluate the pharmacokinetics of CP in other populations (e.g., paediatric population from 1 year old to adolescents) and inflammatory conditions for which CP is indicated.

## 1 INTRODUCTION

Clobetasol propionate (CP) is a potent topical corticosteroid indicated for short‐term treatment (2–4 weeks) of psoriasis (excluding widespread plaque psoriasis), recalcitrant dermatoses (e.g., recalcitrant eczema) and other skin conditions which do not respond satisfactorily to less potent steroids. CP is available as both a cream and ointment formulation (Dermovate, 0.05% w/w). The current recommendations for the use of CP cream or ointment is to apply it thinly over the affected areas once or twice daily, and total weekly doses should not exceed 50 g for 2–4 weeks.^1^, ^2^

As CP belongs to the most potent class of topical corticosteroid (TCS), there is a concern that it may exert adverse systemic effects, including hypothalamic–pituitary–adrenal (HPA) axis suppression and Cushing's syndrome.^3^ However, it is not evident whether such a concern applies to CP cream or ointment when it is used as recommended according to its label. High doses (>50 g/week) or prolonged use (>2 years) in patients with AD or psoriasis have been linked to effects of HPA axis suppression and Cushing's syndrome. In one study, a linear relationship between the dose CP cream with HPA axis suppression was observed, with doses of CP cream above 50 g/week associated with cortisol suppression.^4^ These reports highlight the importance of using CP as recommended, as clinically meaningful HPA suppression was not observed at therapeutic doses.^5^, ^6^

High systemic concentrations of corticosteroids can cause HPA suppression; however, the amount of drug reaching systemic circulation is expected to be low following topical application (<1%) compared to other routes of administration (oral, inhaled, IV).^3^ In fact, high doses of CP cream or ointment (>20 g daily) are required for CP concentrations in plasma to be quantifiable.^7^, ^8^, ^9^, ^10^ In these studies, large variability in systemic concentrations of CP was observed following topical application in subjects with normal skin^10^ and subjects with AD.^7^, ^8^, ^9^

Characterizing the pharmacokinetics of CP in the relevant patient population would provide further insight into the implications of interindividual variability in drug disposition. However, conducting such clinical studies is not feasible or ethical and an alternative approach is required.

Physiologically‐based pharmacokinetic (PBPK) modelling is a mechanistic approach to quantitatively predict the pharmacokinetics of drugs in different populations by considering the biological system and the physiochemical properties of the drug.^11^ The multi‐phase and multi‐layer mechanistic dermal absorption (MPML MechDermA) model in the Simcyp Simulator (Population‐Based PBPK Simulator; Simcyp Division, Certara UK Limited, UK) quantitatively describes drug uptake and permeation through human skin, accounting for formulation characteristics as well as body site‐ and sex‐specific population variability.^12^ By considering physiological differences in the skin barrier properties, this model can be used to extrapolate to different patient populations and determine local and systemic drug exposure. Importantly, the MPML MechDermA model also incorporates information on formulation attributes, physiochemical and structural characteristics and dynamic changes, such as evaporation of volatiles.^12^ This model has been used to quantify local and systemic exposures of various dermatological products.^13^, ^14^

The objective of this study was to develop a PBPK model for CP to describe local and systemic exposure in healthy adult subjects and in patients with AD. The availability of a PBPK model will provide an opportunity to utilize all available clinical data to predict local and systemic CP exposure across different clinical scenarios and conditions to inform current dosing guidelines.

## 2 METHODS

### 2.1 Clinical data

The clinical studies used to develop and verify the PBPK model describing the plasma concentration *vs*. time profiles of CP in the healthy adult population and in patients with AD are shown in Tables 1 and 2, respectively. Blood samples for the evaluation of pharmacokinetics (PK) were collected after intravenous (IV) administration (Study A^15^) and topical application of Dermovate cream (0.05% w/w CP; Study B (part1),^16^ Study B (part 2),^16^ Study C^17^ and Study D (part 1)^18^) and Dermovate ointment formulations (0.05% w/w CP; Study Study D (part 2), and Harding et al. ^10^). The concentrations of CP measured in the stratum corneum (SC) following topical application of the cream formulation^19^ were also used for model development.

**TABLE 1:** Studies on the clinical pharmacokinetics of clobetasol propionate in healthy subjects.

Study	Study details	No. subjects	Population	Dosage regimen	Application sites	PK sampling	Reference
A	Bioavailability study of CP following oral and IV administration	6	Healthy males	2 mg CP administered by intravenous and oral routes.	N/A	Pre‐dose, and at 0.25, 0.5, 0.75, 1, 2, 3, 4, 6, 8, 10, 12, 14, 24, 27, 30 h post‐dose. Urine collections were made over 0–2, 2–4, 4–6, 6–8, 8–12, 12–24 and 24‐30 h post‐dose.	^15^
B (part 1)	PK study and investigation of the percutaneous absorption of CP cream (0.05%) applied to healthy volunteers	8	Healthy males	Priming dose of 30 g CP cream (0.05%) applied 13 h prior to second application of 30 g CP cream (0.05%).	Whole body excluding face, neck, feet and genital area	Pre‐dose, 2, 4, 6, 8, 10, 12, 24, 48 and 96 h post‐dose.	^16^
B (part 2)	PK study and investigation of the percutaneous absorption of CP cream (0.05%) applied to healthy volunteers (multiple dose)	7	Healthy males	15 g once daily for 12 nights	Whole body excluding face, neck, feet and genital area	Pre‐dose, 2, 4, 6, 8, 10, 12, 24, 48 and 96 h post‐dose.	^16^
C	PK study and investigation of the percutaneous absorption of CP cream (0.05%) applied to healthy volunteers (twice daily, multiple dose)	6	Healthy males	12.5 g twice daily of CP cream (0.05%) for 6 days	Trunk (upper torso)	Pre‐dose, and at 12, 60, 120, 168, 180 and 300 h	^17^
D (part 1)	PK study and investigation of the percutaneous absorption of CP cream (0.05%) with and without the use of occlusion	6	Healthy males	25 g CP cream (0.05%) for 13 h. Whole body without occlusion (protected with t‐shirt and long‐john pants), or with occlusion (polythene suit)	Whole body excluding face, neck, feet and genital area	Pre‐dose, 1, 2, 3, 5, 7, 9, 11, 13, 24, 30, 48, 54 h post‐dose	^18^
D (part 2)	PK study and investigation of the percutaneous absorption of CP ointment (0.05%) with and without the use of occlusion	6	Healthy males	25 g CP ointment (0.05%) for 13 h. Whole body without occlusion (protected with t‐shirt and long‐john pants), or with occlusion (polythene suit)	Whole body excluding face, neck, feet and genital area	Pre‐dose, 1, 2, 3, 5, 7, 9, 11, 13, 24, 30, 48, 54 h post‐dose	^18^
Au et al. 2010	Investigation of the concentration of CP in the stratum corneum using tape stripping method	30	Healthy male and female subjects	CP cream (0.05%) dose of 5.5 mg/cm^2^.	2 × 2 cm^2^ application area on inner forearm (volar)	Cream left on skin site for 2 h, excess was removed. Tape striping is done 2 h and 5 min after dose.	^19^
Harding et al. 1985	PK study and investigation of percutaneous absorption of CP from novel ointment and cream formulations (Study 4, ointment only)	8	Healthy males	Priming dose (13 h prior) and a 30 g single application of CP cream (0.05%) to whole body (excluding face, neck, feet and genital area).	Whole body excluding face, neck, feet and genital area	Pre‐dose, 2, 4, 6, 8, 10, 12, 24, 48 and 96 h after application.	^10^

**TABLE 2:** Studies on the clinical pharmacokinetics of clobetasol propionate in subjects with atopic dermatitis (AD).

Study	Study details	No. subjects	Population	Treatment details	Application sites	PK sampling	Reference
Hehir et al., 1983	Investigation of the pharmacokinetics of clobetasol propionate and clobetasol butyrate after a single application of ointment	5	Male and female subjects with eczema	CP ointment (0.05%), 25 g single application	Whole body excluding face, neck, feet and genital area	Pre‐dose, 1, 3, 6, 9, 12, 24, and 48 h after application. Some samples also collected at 36 h, 72 h and 96 h post‐dose (n = 6). Additional patients with sample collected at 36 h and 72 h.	^7^
Sparidans et al., 2010	LCMS assay to quantify clobetasol propionate concentrations in human serum. The study includes PK data from two patients with AD	2	Female subjects with severe AD	CP ointment (0.05%), 30 g twice daily for 7 days followed by either once daily for further 5 days (Subject A, 19 years) or twice daily for a further 10 days (Subject B, 66 years).	Whole body excluding face, neck, feet and genital area. Assumed AD affected 30% of BSA.	Day 1, 2, 9 and 14.	^8^
van Velsen et al., 2012	Investigation of plasma concentrations of clobetasol propionate either one or two applications of clobetasol propionate cream (0.05%) in patients with severe AD.	25	Male and female subjects with severe AD with mean BSA affected by disease of 59%	CP ointment (0.05%), 20–30 g receiving either one dose or two doses.	Whole body excluding face and genital area. Assumed AD affected 59% of BSA.	17 h post‐dose for subjects receiving one dose of clobetasol and 14 h post‐dose for subjects receiving two doses of clobetasol.	^9^

Most of the studies used to develop and verify the dermal PBPK model included pharmacokinetic data following whole body applications (Study B (part 1),^16^ Study B (part 2),^16^ Study C,^17^ Study D (part 1),^18^ and Study D (part 2)^18^). As there was limited information on the application site and information on the application area was not available, assumptions were made based on the mean body surface area (BSA) of the population and the surface area of each body site was estimated using the rule of nines.^20^ The trial design for each study following topical application of the CP cream and ointment formulation is shown in Tables S4 and S5. The trial design for the adult population with AD following topical application of the CP ointment formulation (0.05%, w/w) is shown in Table S6. Published pharmacokinetic data from publications were digitized using WebPlotDigitizer.^21^

### 2.2 PBPK model development

**FIGURE 1:**
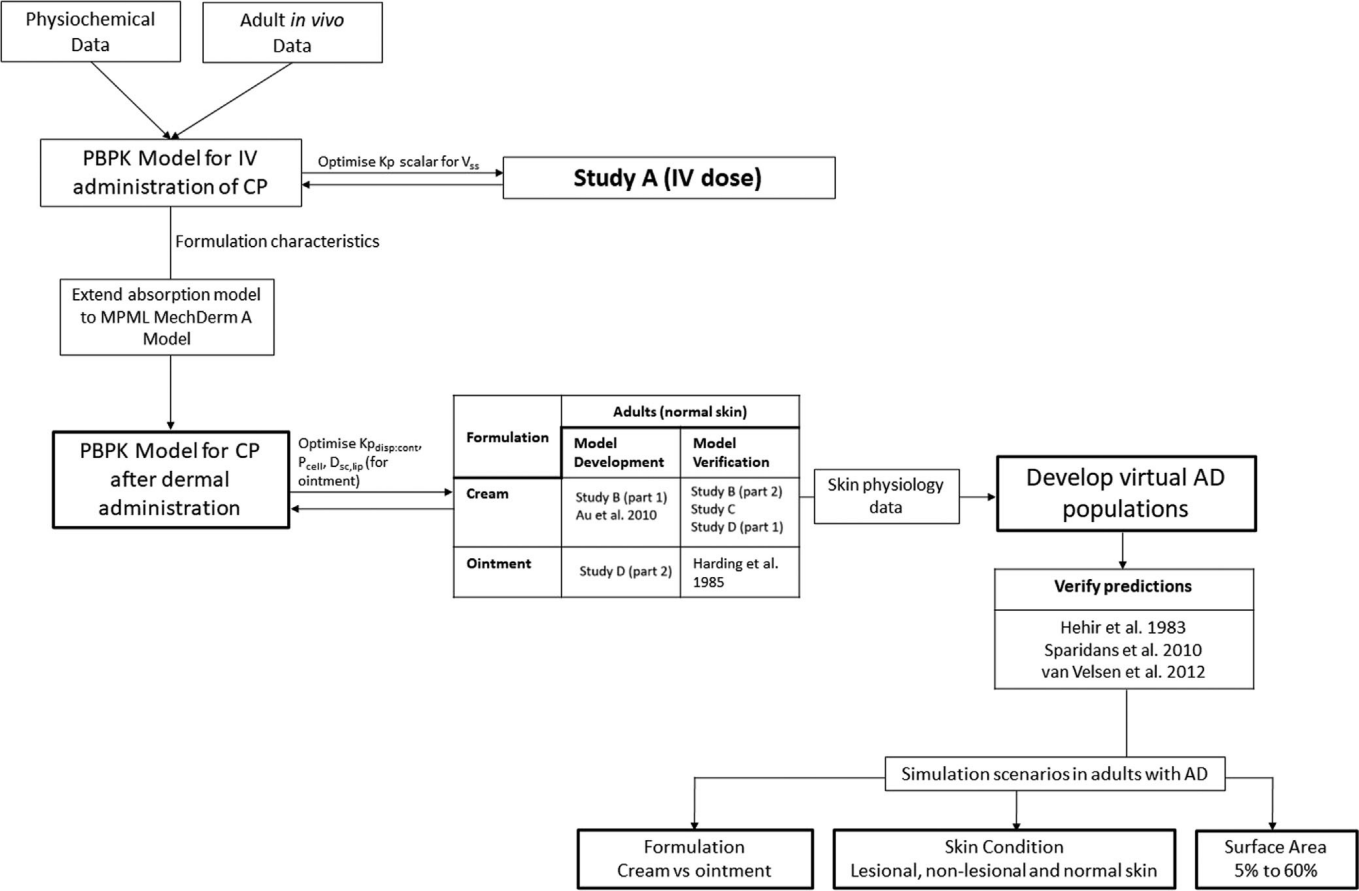
PBPK workflow for the development and verification of the model performance for clobetasol propionate cream and ointment formulations.

The Simcyp Simulator population‐based PBPK software (Certara UK Ltd., Simcyp Division, Sheffield, UK; Version 22) was used to develop the PBPK model. The strategy for developing the clobetasol PBPK model followed best practices and utilized a ‘learn and confirm’ approach^22^, as shown in Figure 1. The Simcyp R library package was used to simulate different clinical trial scenarios, aimed at exploring the implications of formulation, skin disease and surface area on systemic and tissue exposure.

#### 2.2.1 Intravenous administration

The Simcyp compound file for CP was developed using physicochemical properties of the drug obtained from the literature and from the DrugBank database (https://go.drugbank.com/). The mean parameter values of systemic clearance (CL_IV,_ 27.6 L/h) and renal clearance (CL_R_, 2 L/h) from Study A (*n* = 6) following an IV dose were used as initial estimates for the PBPK model. These pharmacokinetic parameters were determined previously using a two‐compartment model, with an initial volume of distribution of about 18 L and a post‐distributive volume of distribution averaging 164 L (Study A, Table 1).

A full PBPK distribution model was used, which enables concentrations to be simulated in various major body organs. The steady‐state volume of distribution (V_ss_), which represents the predicted volume in blood and individual tissues of the PBPK model, and the tissue‐plasma partition coefficient (K_p_) was predicted using the Rodgers and Roland Method.^23^ The K_p_ scalar was optimized to match the V_ss_ prediction with observed values using Study A (164 L). To assess the accuracy of the predicted concentrations following oral administration of CP, the model was extended to a first‐order absorption model and pharmacokinetic predictions were compared with observed concentrations (Tables S1 and S2 and Figure S1).

#### 2.2.2 Dermal administration

The dermal absorption of CP was simulated by using the MPML MechDermA model in Simcyp (Simcyp Simulator V22), which includes eight components: (1) formulation; (2) stratum corneum, as a multi‐layer multi‐phasic (lipid, protein and water) structure; (3) viable epidermis; (4) dermis; (5) hair follicle; (6) subcutis; (7) muscle (deep tissue) and (8) local vasculature (blood circulation) (Figure S2).

##### Formulation characteristics

The physicochemical and structural characteristics of the CP cream and ointment formulations were incorporated into the PBPK model and are shown in Table 3. The topical CP formulations (cream and ointment) were simulated as biphasic systems (oil‐in‐water) with mean dispersed phase droplets of 10.5 μM. The composition of CP cream used for model development was reported previously and consisted of propylene glycol, water, various emulsifying agents, pH modifiers, preservatives and buffer agents.^24^, ^25^ The CP ointment formulation consists of propylene glycol, sorbitan sesquioleate and white soft paraffin.^2^ Some of the key parameters estimated using the MPML MechDermA model require knowledge of the solubility of CP in the dispersed and continuous phase of the formulation. As CP is lipophilic and insoluble in water (3.86 μg/mL^26^), the continuous phase of the formulation contains propylene glycol (PG, 47.5% of the total cream composition^25^), which is a penetration enhancer and facilitates the diffusion of CP into the SC. Although the solubility of CP was determined previously only in aqueous solutions of PG and water, its solubility is in the presence of the other excipients was assumed to be similar to that in PG.^25^

**TABLE 3:** Characteristics of CP cream and ointment (0.05%) used for the development of a dermal PBPK model.

Parameter	Cream	Ointment	Source
Formulation simulation option	Emulsion	Emulsion	
Viscosity (cP)	2773	6249	GSK Internal Data
pH of formulation	5	5	GSK Internal Data
Density of formulation (g/cm^3^)	1.1	0.8	GSK Internal Data
Drug solubility in continuous phase (mg/mL)	0.63	1.09	Based on CP solubility data for 70% PG/30% water (cream) and 80% PG/20% water (ointment)^25^
Intrinsic solubility in water (mg/mL)	0.00387	0.00387	CP solubility in water
Initial drug amount ratio dispersed/continuous phase	5.641	2.222	QSAR Predicted
Dispersed : Continuous Kp (Kp_disp:cont_)	20	20	Optimized using in vivo data (Study B,^16^)
Volume fraction of dispersed phase (%)	22	10	Based on formulation composition
Radius of dispersed phase droplets (μM)	10.5	10.5	Mean size based on GSK Internal Data
Droplet permeability (cm/h)	1 x 10^−5^	1 x 10^−5^	Default
Evaporation profile	Zero order	No evaporation considered	Evaporation profile for cream was calculated based on water content and volatile vehicle characteristics of water
Molecular weight of vehicle (g/mol)	52.1		
Density of vehicle (g/ml)	1.03		
Zero order evaporation rate (ml/h)	31.69 (30% CV)		
Vapour pressure at skin temperature (mm Hg)	9.87 (30% CV)		
Maximum %(v/v) vehicle evaporated	30% (30% CV)		
Air velocity (m/sec)	0.5 (30% CV)		Default value

The metamorphosis of formulation due to evaporation was considered for the cream formulation but not for the ointment formulation. CP cream contains 30% water content^27^ and a mass reduction of 30% was observed at 5 h for Dermovate cream when stored at room temperature.^28^ Since CP ointment did not have any volatile components, it was assumed this formulation is not affected by evaporation.

##### Optimizing dermal absorption parameters

The parameters describing the partition and diffusion through the layers of the skin were initially all predicted using built‐in quantitative structure–activity relationship (QSAR) models, but this resulted in poor predictions of CP in plasma and SC. A global sensitivity analysis was conducted to determine the parameters that influence the local and systemic concentrations of CP. The following parameters were found to influence the local and systemic PK of CP: the partitioning coefficient for the dispersed : continuous phase (Kp_dis:cont_), the corneocyte permeability (P_cell_), the SC lipid to vehicle partition coefficient (Kp_SClip:v_) and the diffusion coefficient of the drug through the SC (D_SC,lip_). A description of these parameters and their effects are shown in Table 4.

**TABLE 4:** Description and effect of MPML MechDermA parameters on the pharmacokinetic characteristics of clobetasol propionate.

Parameter	Description	Effect on PK
P_cell_	Corneocyte membrane permeability: this parameter indicates the extent of partitioning of the drug into the corneocytes in the SC. There are no QSARs available to predict this parameter.	The default value of P_cell_ is 1 × 10^−5^ (cm/h), and the lowest value is 1 × 10^−12^. A low number indicates no partitioning into the corneocytes, therefore there is no drug accumulation in the skin. A high number will indicate high partitioning and accumulation into the corneocytes and drug will stay in the SC over a prolonged period of time. This parameter was optimized to 1 × 10^−7^ (cm/h) using Study A and Au et al.^19^
Kp_SClip:v_	SC Lipid:vehicle partition coefficient. Kp_SClip:v_ controls the partitioning of the drug into the first layer of the SC and is rate‐limiting. This parameter is dependent on the solubility of CP.	A lower number indicates a slower rate of permeation into SC. This parameter was calculated by using the predicted partitioning Ksclip:water (value = 292), which describes partitioning between water and SC lipids, with the solubility ratio of CP in the continuous phase:water (0.633/0.0086 mg/mL = 164.6). Therefore this resulted in a KpSClip:v value of 1.77 (292/164.6).
D_SC,lip_	SC lipid diffusion coefficient. This parameter controls the diffusion of the drug through the SC and the intercellular pathway is assumed to be the major route for most drugs.^12^	For the cream formulation, this parameter was predicted using Johnson QSAR equation^29^ (3.03 × 10^−4^ cm^2^/h). For the ointment formulation, this value was optimized to a value of 7.0 × 10^−3^ cm^2^/h, to indicate a higher rate of permeation through the SC due to a more hydrated skin.
Kp_dis:cont_	The partitioning coefficient of CP for the dispersed : continuous phase of the formulation. This parameter describes the solubility of the drug in the different phases and is important for defining initial conditions.^12^	The solubility of CP in the different phases of the formulation is unknown and this value can was tested in scenarios with values between 3.65 and 32.^20^ For Simcyp Simulator V22, this was a user input parameter and cannot be calculated. Therefore, this parameter was optimized to a value of 20, which provided the best fit for the local and systemic concentrations of CP.

The P_cell_ and Kp_dis:cont_ parameters are user‐defined and there are no equations available to predict the parameter value.^12^ The P_cell_ parameter describes the partitioning of the drug into the corneocytes, which impacts the extent of drug accumulation in the SC. Kp_dis:cont_ describes the solubility of CP in the different phases and is important for defining the initial conditions of the amount of drug in the dispersed/continuous phase. As there are no QSAR equations to predict P_cell_ and Kp_dis:cont_, these parameters were optimized using pharmacokinetic data in plasma and in SC (Study B).^19^ Kp_SClip:v_ was predicted using QSAR equations based on the solubility of CP in the different phases of the formulation.

To improve predictions for the ointment formulation, D_SC,lip_ was optimized to a higher value to indicate increased permeation of CP due to a more hydrated skin. Study D (part 2) was used to optimize D_SC,lip_.

##### Verifying dermal absorption parameters

The optimized model parameters were verified using separate studies to ensure the model can be generalized to other population groups. Studies B (part 2), C and D (part 1) were used to verify the PBPK model for the cream formulation. A separate study was used to verify the predictions for the ointment formulation.^10^

##### Effect of occlusion

The effect of occlusion, using a polythene (PE) suit, was investigated in Study D (part 1 and 2). It was assumed that the main effect of occlusion was to prevent the evaporation of the cream formulations and for increased permeation due to a more hydrated skin. Therefore, predictions of CP in plasma with and without occlusion were evaluated by removing the effect of evaporation and by setting the D_SC,lip_ parameter to the same value as the ointment formulation.

### 2.3 Developing a skin barrier impairment model and lesion model in the AD population

To predict the systemic exposure to CP in the population of interest, a model for skin barrier impairment was required. To develop this model, changes in the skin physiology parameters for the virtual healthy subject population (Sim‐Healthy Volunteer) were made based on observed changes in skin barrier function, hydration, pH and corneocyte size for patients with AD (Table 4). Lesional skin is defined as skin that is affected by AD with marked inflammation whilst non‐lesional skin is defined as clinically normal appearing skin in patients with AD.^30^, ^31^ Non‐invasive bioengineering methods were used to evaluate the skin barrier function, including measurement of the transepidermal water loss (TEWL) and hydration of the SC (corneometry). TEWL is a parameter that has been used extensively to evaluate the skin barrier function and was found to be associated with the skin thickness.^32^ Measurements of TEWL in adult subjects with AD were found to be higher for both non‐lesional and lesional skin.^31^, ^33^ Since the skin thickness (SC, viable epidermis, dermis) is the parameter most sensitive to the dermal absorption of CP, the reduction in skin thickness (%) relative to subjects with normal skin was calculated based on the mean TEWL values for lesional and non‐lesional skin (Table 5). The same method was used to calculate reduction in skin hydration and increase in pH relative to subjects with normal skin. In adults with AD, corneocyte size was also found to be approximately 30% smaller compared to normal skin.^34^ A sensitivity analysis was conducted to explore the effect of each parameter modification on the plasma concentrations of CP (Table S3).

**TABLE 5:** Changes to skin parameter values relative to normal skin.

AD population	Skin condition	Reduction in skin Thickness^a^ (%)	Reduction in skin Hydration^b^	Increase in pH^c^ (%)	Reduction in corneocyte surface area^d^ (%)	References
**Adults**	Lesional skin	80	55	15	30	^31^, ^33^, ^34^
	Non‐lesional skin	26	36	5	No data	

^a^ The reduction in skin thickness refers to a reduction in SC layers, viable epidermis and dermis thickness, which varies by body site. For normal skin (abdomen site), the mean number of SC layers is 13, mean viable epidermis thickness is 50 μM and mean dermis thickness is 2114 μM.

^b^ For normal skin (abdomen site), the mean hydration level for SC for normal skin (% water volume) is 33.9% for top 25% of SC layers, 44.7% for upper middle 25% of SC layers, 55.5% for lower middle 25% of SC layers and 66.4% for bottom 25% of SC layers.

^c^ For normal skin (abdomen site), the mean skin surface pH for SC is 5.6.

^d^ For normal skin (abdomen site), the mean corneocyte length is 39.8 μM and the width is 33 μM.

### 2.4 Verifying the AD population

To evaluate PBPK predictions for adults with AD, three clinical studies with PK data were used^7^, ^8^, ^9^ for model verification purposes.

In one study, it was observed that subjects with higher BSA% affected by AD have higher systemic exposure to CP.^7^ In this study, all subjects with AD received the same dose (25 g, applied to the whole body); however, the surface area affected by AD ranged from 40% to 95% (% of total BSA). To simulate this scenario, the total application area and the total dose (therefore application thickness) was kept the same for each subject. To simulate the changes in surface area affected by AD, the proportion of lesional to non‐lesional skin varied based on the %BSA affected by the disease. For example, for a subject with 40% BSA affected, it was assumed that 40% of the total application area was applied to the lesional skin and the remaining 60% was applied to non‐lesional skin. The application of CP to non‐lesional skin is a modelling concept to mimic the dosing of CP in this study. In clinical practice, CP would only be applied to lesional skin. Simulation scenarios covering a range of %BSA affected by AD (5%–95%) were performed using a 10 subject × 10 trial design.

For the remaining studies, individual data were not available and the mean BSA affected by AD (30%^8^, 59%^9^) was considered in the simulations.

### 2.5 Model performance and evaluation

Whenever possible, a fixed trial design was used to match the demographics of the simulated population with the clinical trial population. If this was not possible, the trial simulation included the total number of subjects involved in a clinical study, the proportion of female subjects and the age range of the clinical study. The number of simulation trials was such that at least 100 subjects were simulated to compare with the observed data (Tables S4, S5 and S6).

Model performance was assessed by using goodness‐of‐fit plots by overlaying the observed and simulated mean concentration–time profiles with the 5^th^ and 95^th^ percentiles. The variability in the data was shown using error bars (95% CI). The predicted/observed ratio was also determined for the secondary pharmacokinetic parameters (*C*_max_, AUC). The acceptance criteria assume that model performance is acceptable if the predicted/observed ratio remains within 0.5–2.0 for each parameter.

### 2.6 Characterizing absorption, local and systemic exposure to CP

The final PBPK model was used to simulate local and systemic exposure for adults with varying %BSA affected by AD. To determine the dose to be used, the fingertip unit (FTU) guidance was used.^35^ In this guidance, 1 FTU is equivalent to 0.5 g of topical corticosteroid.^35^ One adult leg and foot involvement (19% of BSA based on the rule of nines^20^) would require 8 FTU (4 g) of topical corticosteroid.^35^ Assuming the mean BSA of an adult is 1.8 m^2^, the recommended dose based on this guidance is about 1.2 mg/cm^2^.

For this simulation scenario, adults with AD (5%–60% of BSA affected) were treated with CP cream or ointment (0.05% w/w) at a dose of 1.2 mg/cm^2^ for 2 weeks. The simulations were performed on lesional skin, non‐lesional skin and normal skin. A 10 subject × 10 trial design was used, with subjects aged 18–80 years, and equal proportion of male and female subjects. The abdomen was used as the representative site.

## 3 RESULTS

### 3.1 Intravenous administration

**FIGURE 2:**
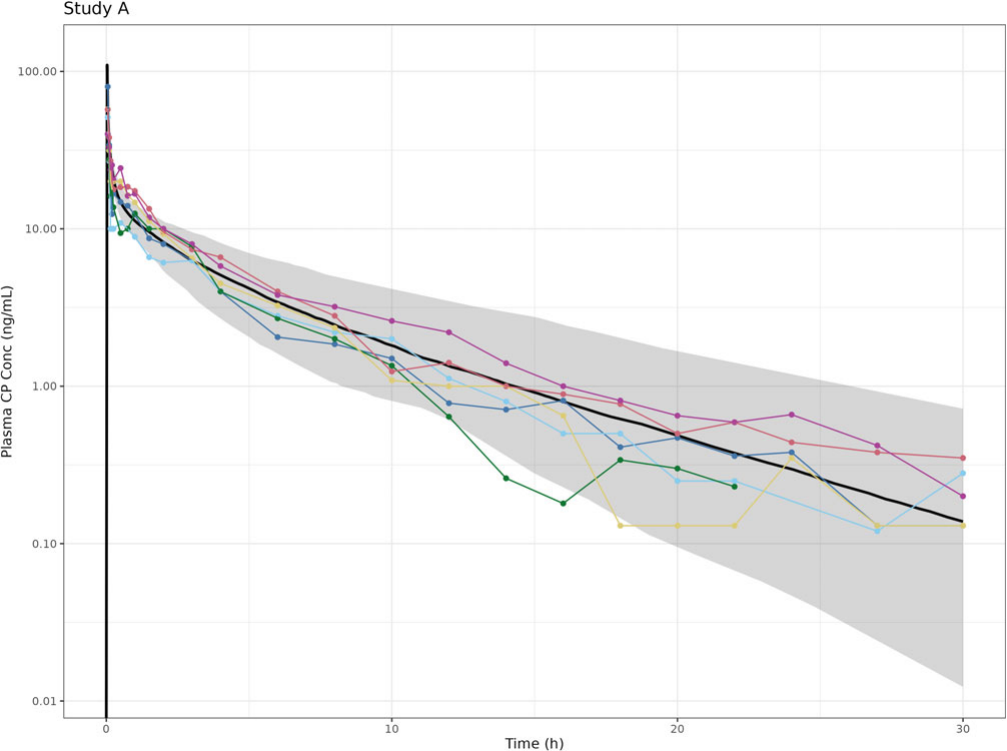
CP plasma concentration–time profile following IV administration of 2 mg to six healthy adult subjects. Black line represents the mean of the simulated clobetasol concentrations, grey shaded area represents the 5^th^ and 95^th^ percentiles of simulated clobetasol concentrations, the points represent the observed concentrations.

The physicochemical, distribution and elimination parameters for the IV model are shown in Table 6. There was good agreement between the observed and predicted concentrations (Figure 2) and the predicted/observed ratios of PK parameters were within the acceptance criteria (range 0.92–1.33; Table 7). The PBPK model was also used to predict the PK of clobetasol following a single oral dose of 2 mg using a first‐order rate of absorption model. There was good agreement between the observed and predicted concentrations (predicted/observed ratio ranged from 0.80–1.29) following oral dosing, and the oral bioavailability was estimated to be 50% (see Figure S1 and Table S2).

**TABLE 6:** PBPK model parameters for clobetasol propionate.

	Parameter	Value	Source
**Physicochemical properties**	Molecular weight (g/mol)	467	https://go.drugbank.com/drugs/DB01013
	Log P (o/w)	3.5	
	pKa	13.63	
	Compound type	Neutral	
	Blood to plasma ratio	1.2	QSAR predicted
	fu_p_	0.07	QSAR predicted
**Absorption**	Absorption model	MPML MechDermA	
	Drug Partition Coefficients (K)		
	K_sclip/water_	292	Hansen 2013^36^
	K_sclip/vehicle_	1.77	Hansen 2013^36^
	K_Sebum/Water_	580	QSAR predicted
	K_SC/VE_	4.26	Shatkin and Brown 1991^37^
	K_Dermis/VE_	0.089	Modified Chen 2015^38^
	K_Dermis/Sebum_	0.015	QSAR predicted^12^
	K_Dermis/Blood_	2.776	Shatkin and Brown 1991^37^
	K_Subcutis/Dermis_	1	Assumed^a^
	K_Muscle/Subcutis_	1	Assumed^a^
	K_Muscle/Blood_	1	Assumed^a^
	K_Subcutis/Blood_	1	Assumed^a^
	Diffusion coefficients (cm^2^/h)		
	D_sclip_ (cream)	3.03 x 10^−4^	Johnson 1996^29^
	D_sclip_ (ointment)	7.0 x 10^−3^	Optimized using Study D (part 2)
	Tortuosity of lipid diffusion pathway of SC	2335	Johnson 1996^29^
	D_ve_	3.03 x 10^−4^	Modified Chen 2015^38^
	D_Dermis_	3.03 x 10^−4^	Modified Chen 2015^38^
	D_Sebum_	9.70 x 10^−5^	Modified Chen 2015^38^
	Fraction unbound in SC (fu_sc_)	0.11576	Polak and Patel 2016^39^
	P_cell_	1 x 10^−7^	Optimized using in vivo data (Study B, Au et al.^19^)
	D_Subcutis_	3.03 x 10^−4^	Assumed similar to the dermis
	D_Muscle_	3.03 x 10^−4^	Assumed similar to the dermis
**Distribution**	Distribution model	Full PBPK	
	V_ss_ (L/kg)	2.34	Rodgers and Rowland^23^ (Simcyp Simulator, Method 2)
	K_p_	0.22	Optimized using Study A
**Elimination**	Clearance type	In vivo Clearance	
	CL_IV_ (L/h)	27	CL_IV_ from Study A
	CL_R_ (L/h)	0.12	Study A

Abbreviations: CL_IV_, systemic clearance; CL_R_, renal clearance; fu_p_, fraction unbound in plasma; K_p_, partition coefficient; Log P, logarithm of the octanol–water partition coefficient (lipophilicity); pKa, negative logarithm of the acid dissociation constant; V_ss_, volume of distribution at steady‐state.

^a^ Parameters are not sensitive to simulation outcomes.

**TABLE 7:** Observed and predicted secondary pharmacokinetic parameters following administration of clobetasol propionate to healthy male subjects.

Study	Formulation	Dose (g)^a^	Dosing regimen	*n*	Parameter	Observed	Predicted	Ratio (Pred/Obs)
						Mean (95% CI)	Mean (95% CI)	
Study A	IV	0.02	Single dose	6	*t*_1/2_ (h)	3.9 (0.2)^b^	5.2 (4.9–5.6)	1.33
					AUC_0‐30h_ (ng.h/mL)	73.3 (5.7)^b^	78.2 (74.2–82.3)	1.07
					AUC_0‐INF_ (ng.h/mL)	74.8 (6.2)^b^	80.1 (75.8–84.6)	1.07
					CL_IV_ (L/h)	27.6 (2.16)^b^	26.2 (23.7–26.5)	0.95
					CL_R_ (L/h)	0.12 (0)^c^	0.11 (0.11–0.11)	0.92
Study B (part 1)	Cream	30	Two applications	7	*T*_max_ (h)^d^	21.0 (12.5–23)	31.1 (27.3–44.7)	1.48
					*C*_max_ (ng/mL)	0.82 (0.50–1.14)	0.68 (0.65–0.71)	0.83
					AUC_13–96_ (ng.h/mL)	23.3 (7.45–40.8)	42.9 (40.3–45.5)	1.84
Study B (part 2)	Cream	15	Once daily, multiple dose	7	*C*_max_ (ng/mL)	0.76 (0.38–1.14)	0.85 (0.79–0.90)	1.12
					AUC_0–277_ (ng.h/mL)	108 (38.8–178)	195 (183–208)	1.81
Study C	Cream	12.5	Twice daily, multiple dose	6	AUC_0–300_ (ng.h/mL)	200 (137–263)	129 (114–144)	0.65
Study D (part 1)	Cream	25	Single application	6	*T*_max_ (h)^d^	13.0 (7.0–24.0)	20.8 (14.6–35.1)	1.60
					*C*_max_ (ng/mL)	0.32 (0.15–0.48)	0.36 (0.34–0.38)	1.13
					AUC_0–54_ (ng.h/mL)	9.07 (1.81–16.5)	15.3 (14.5–16.1)	1.67
Study D (part 2)	Ointment	25	Single application	6	*T*_max_ (h)^d^	8.0 (3.0–13.0)	19.8 (14.3–34.02)	2.47
					*C*_max_ (ng/mL)	0.31 (0.18–0.49)	0.30 (0.28–0.32)	0.97
					AUC_0–54_ (ng.h/mL)	8.20 (1.31–15.2)	13.3 (12.4–14.1)	1.62
Harding et al. 1985^e^	Ointment	30	Two applications	8	*T*_max_ (h)^d^	21.0	30.0 (26.7–45.8)	1.43
					*C*_max_ (ng/mL)	0.62	0.83 (0.77–0.89)	1.34
					AUC_13–109_ (ng.h/mL)	31.5	52.7 (48.3–57.1)	1.67

Abbreviations: AUC, area under the plasma concentration–time curve; *C*_max_, peak plasma concentration; CL_P_, total plasma clearance; CL_R_, renal clearance; *t*_1/2_ apparent half‐life of elimination.

^a^ The topical formulations (cream and ointment) contain 0.05% w/w CP. Therefore, the dose refers to the amount of drug product and not the amount of CP.

^b^ Observed pharmacokinetic parameters following IV dosing were reported as mean (standard error).

^c^ Only the mean observed data were available.

^d^ *T*_max_ reported as median and range.

^e^ Observed pharmacokinetic parameters based on a compartmental analysis of the data.

### 3.2 Dermal administration

Using two clinical studies which contained local and plasma concentration data (Study B),^19^ P_cell_ was optimized to a value of 1 × 10^−7^ cm/h. Using sensitivity analyses (Study B),^19^ Kp_dis:cont_ was optimized to a value of 20, to indicate a slower rate of permeation of CP into the SC. For the CP cream formulation, the QSAR‐predicted value for the D_SC,lip_ parameter (3.03 × 10^−4^ cm^2^/h) was used. For the ointment formulation, this parameter was optimized to a higher value (7.0 × 10^−3^ cm^2^/h). The final parameter estimates for the dermal PBPK model of CP is shown in Table 5.

**FIGURE 3:**
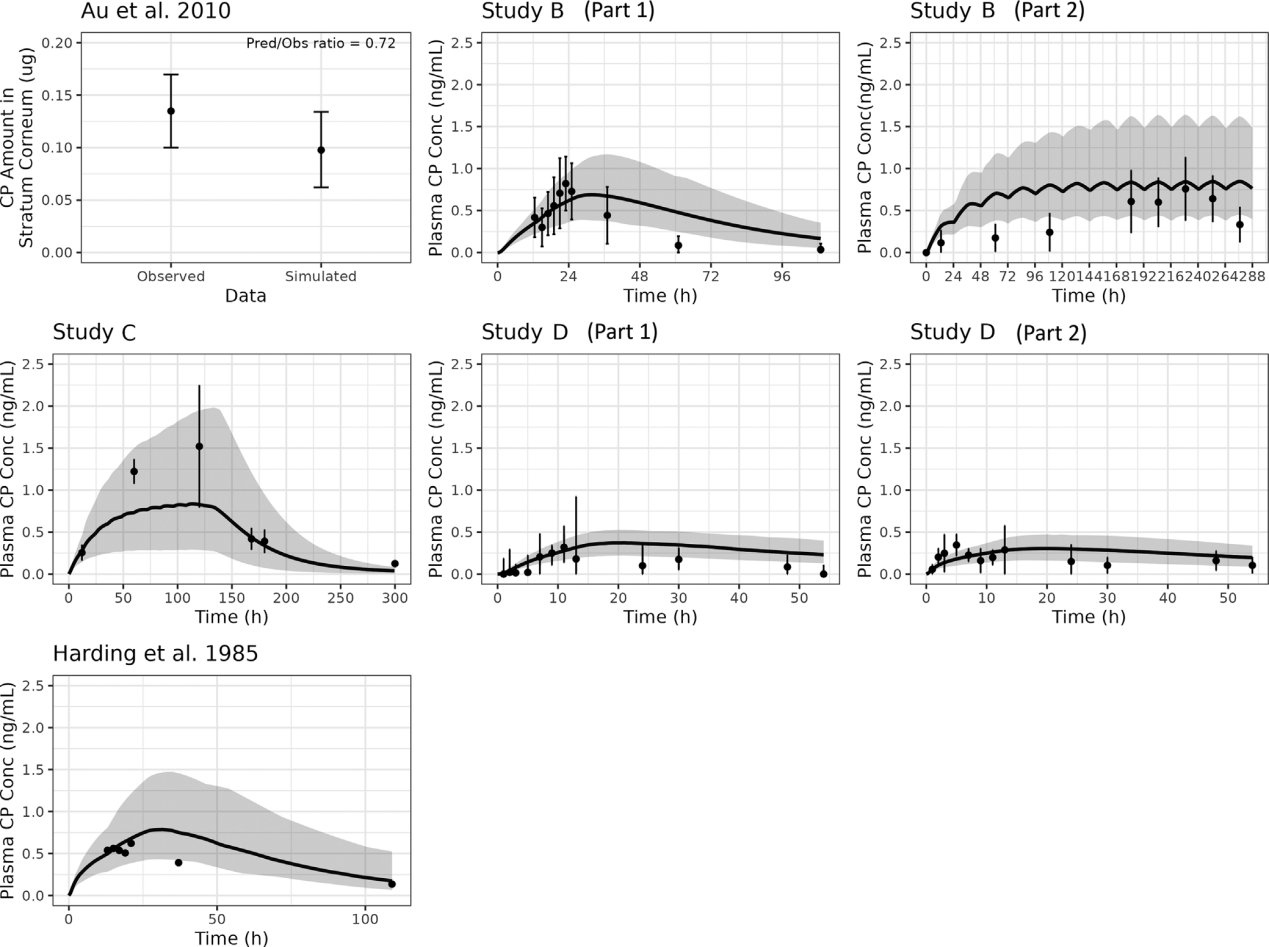
Observed and predicted CP concentration in stratum corneum and plasma following topical application of either the cream (Au et al., 2010,^19^ Study B (part 1), Study B (part 2), Study C, Study D (part 1)) or the ointment formulation (Study D (part 2) and Harding et al., 1985^10^). Black line, the mean of the simulated clobetasol concentrations; grey shaded area, the 5^th^ and 95^th^ percentiles of simulated clobetasol concentrations; points, mean observed concentrations; error bars, 95% CI.

The performance of the dermal PBPK model was evaluated using goodness‐of‐fit plots of the observed and predicted CP concentration–time profiles in SC and plasma following topical application (Figure 3). The predicted/observed ratio of all studies were within the range of the acceptance criteria (Table 7). The apparent plasma half‐life was four‐fold longer following dermal administration (mean 20.8 h) compared to IV administration (mean 5.2 h, Table S7).

**FIGURE 4:**
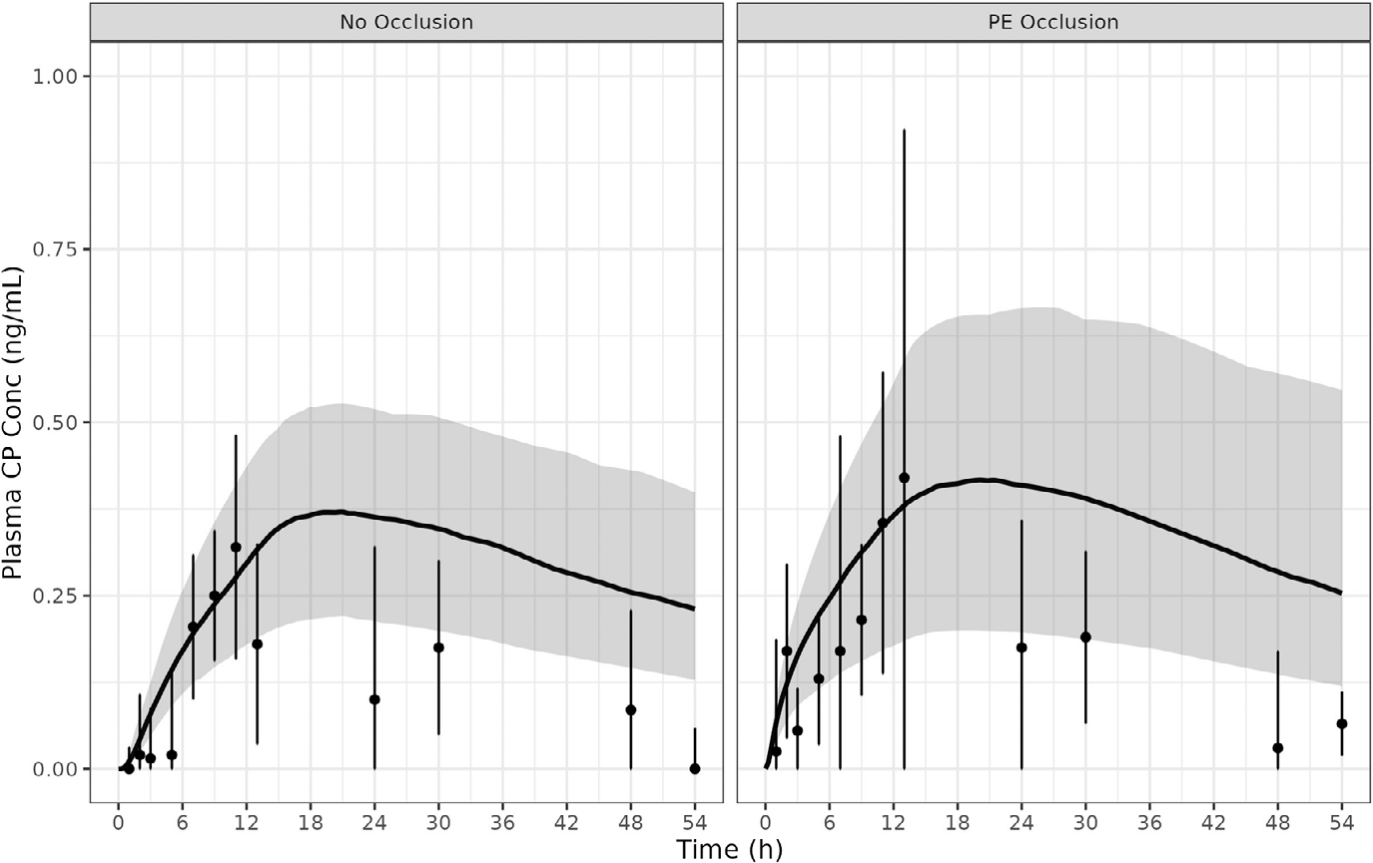
Observed *vs.* predicted CP concentration–time profile following topical application of CP ointment formulation to healthy adults with and without the use of occlusion. Black line, the mean of the simulated clobetasol concentrations; grey shaded area, the 5^th^ and 95^th^ percentiles of simulated clobetasol concentrations; points, mean observed concentrations; error bars, 95% CI.

PE occlusion enhanced the absorption of CP following topical application of the cream formulation, but it had no effect on the ointment formulation. This is consistent with the formulation composition as the cream formulation contains volatile components (30% water), while the ointment formulation does not. The effect of occlusion with CP cream application was modelled by removing the effect of evaporation and setting the D_SC,lip_ parameter value to the same value as the ointment formulation (7.0 × 10^−3^ cm^2^/h), which resulted in a ~20% increase in the AUC_0–54_ (Figure 4, Table 8).

**TABLE 8:** Observed and predicted secondary pharmacokinetic parameters following topical administration of CP cream (0.05% w/w) to healthy male subjects with and without the effect of occlusion.

Study	Occlusion	*n*	Parameter	Observed	Predicted	Ratio (Pred/Obs)
				Mean (95% CI)	Mean (95% CI)	
Study D (part 1)	No occlusion	6	*C*_max_ (ng/mL)	0.32 (0.16–0.48)	0.37 (0.35–0.39)	1.16
			AUC_0–54_ (ng.h/mL)	9.07 (3.09–15.05)	15.3 (14.5–16.1)	1.67
	Polythene occlusion (suit)	6	*C*_max_ (ng/mL)	0.42 (0.14–0.92)	0.42 (0.39–0.45)	1.0
			AUC_0–54_ (ng.h/mL)	12.5 (4.90–20.1)	18.5 (17.3–19.8)	1.48

### 3.3 Predictions of systemic CP exposure in AD population using a skin barrier impairment model

**FIGURE 5:**
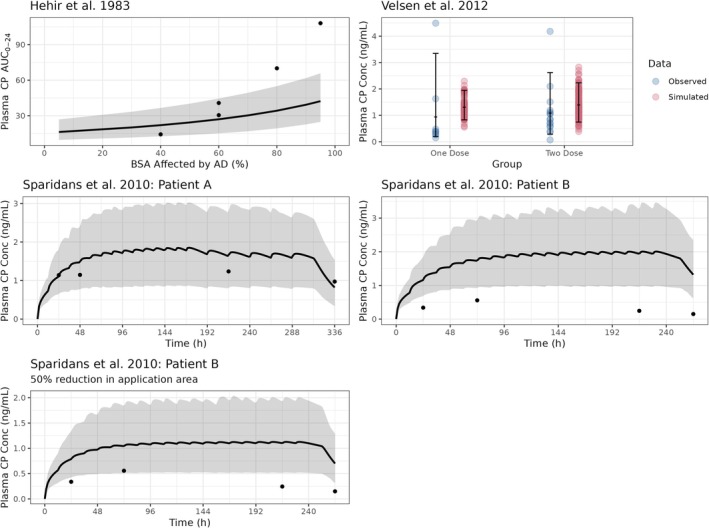
Observed *vs.* predicted CP concentration–time profile following a single application of CP ointment (0.05% w/w) formulation in adults with atopic dermatitis (AD). Points: observed CP concentrations in subjects with AD; solid line, median simulated CP concentrations or AUC_0–24_ (Hehir et al. 1983^7^); shaded area, 5^th^ and 95^th^ percentiles of simulated exposures.

The performance of the skin barrier impairment model was verified using observed CP concentrations in plasma of patients with AD (Figure 5). In one study, there was a proportional increase in the systemic CP exposure (AUC_0–24_) with increasing %BSA affected by AD (Figure 5, Hehir et al.).^7^ Even though the predictions for adults with 40% and 60% BSA affected were well predicted, the CP exposure was underpredicted for patients with 80% and 95% BSA affected by AD.

The virtual AD population was verified using two other clinical studies.^8^, ^9^ The predicted/observed ratio following one (1.39) or two doses (1.29) of CP ointment formulation were within the acceptance criteria for a population with mean BSA affected by AD of 59%.^9^ One study reported two cases (Patient A and Patient B) of AD and it was assumed that 30% of the BSA was affected by AD.^8^ For Patient A (19 year old, female; whole body dose, twice daily for 10 days then once daily for 5 days), the predictions of systemic exposure over the treatment duration (AUC_0–336_), were satisfactory (predicted/observed ratio, 1.48). For Patient B (66 year old, female), despite receiving higher doses of CP (whole body application, twice daily for 14 days) compared to Patient A, the systemic exposure for Patient B was much lower compared to Patient A (Figure 5). The model therefore overpredicted the observed concentrations by five‐fold. Additionally, another scenario was simulated assuming a 50% reduction in the application area (Figure 5). It is likely that Patient B had poor adherence to treatment with CP ointment.

### 3.4 Clinical trial simulations of local and systemic CP exposure considering formulation, skin condition and surface area

**FIGURE 6:**
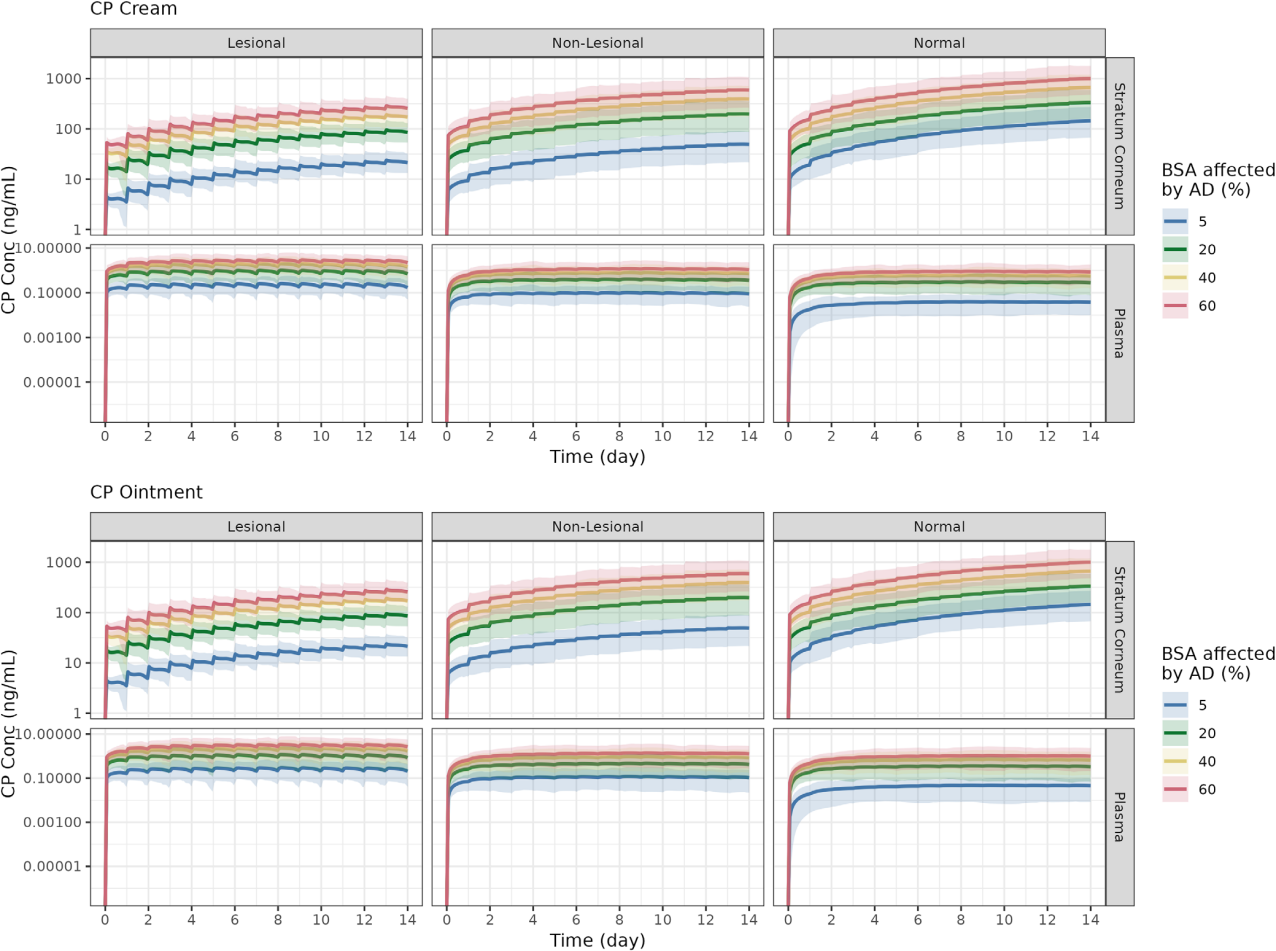
Simulated local and systemic CP concentration–time profile of CP following cream or ointment (0.05% w/w) application, stratified by %BSA affected by AD and skin condition. Solid line, median simulated CP concentrations; shaded area, 5^th^ and 95^th^ percentiles of simulated concentrations.

The final PBPK model was used to simulate local and systemic CP exposure following topical application of either the cream or ointment formulation, over a range of skin conditions (lesional, non‐lesional and normal) and over a range of surface areas (5%, 20%, 40% and 60%; Figure 6). The CP concentration–time profiles for the cream and ointment formulation were comparable (Table 9).

**TABLE 9:** Simulated local (stratum corneum, SC) and systemic (plasma) exposure following topical application of CP cream or ointment formulation (0.05%, w/w) on lesional, non‐lesional and normal skin by application area.

Application area (% BSA)	Daily dose (g)	Weekly dose (g)	Site	CP cream			CP ointment		
				Mean AUC_Day14_ (h.ng/mL) (95% CI)			Mean AUC_Day14_ (h.ng/mL) (95% CI)		
				Lesional	Non‐Lesional	Normal	Lesional	Non‐Lesional	Normal
5	1.1	7.7	SC	527 (496–558)	1163 (1067–12 569)	3350 (3072–3628)	527 (496–558)	1162 (1067–1259)	3350 (3072–3628)
			Plasma	5.2 (4.6–5.8)	2.2 (1.9–2.5)	0.9 (0.8–1.1)	6.1 (0.52–7.1)	2.6 (2.2–3.1)	1.1 (0.8–1.3)
20	4.3	30.1	SC	2113 (1988–2238)	4653 (4269–5037)	7773 (7129–8417)	21 114 (1989–2239)	4653 (4269–5037)	7774 (7129–8418)
			Plasma	20.7 (18.4–23.0)	8.86 (7.69–10.0)	6.85 (5.9–7.8)	24.4 (20.7–28.1)	10.5 (8.6–12.3)	8.1 (6.6–9.6)
40	8.6	60.2	SC	4239 (3989–4489)	9310 (8542–10 078)	15 550 (14 262–19 839)	4243 (3993–4493)	9311 (8543–10 080)	15 552 (14 264–16 840)
			Plasma	41.3 (36.8–45.7)	17.7 (15.4–20.0)	13.7 (11.8–15.6)	48.4 (41.2–55.6)	20.9 (17.2–24.5)	16.1 (13.2–19.1)
60	13.0	91	SC	6377 (6001–6753)	13 971 (12 819–15 124)	23 332 (21 399–25 265)	6385 (6009–6761)	13 974 (12 821–15 127)	23 334 (21 401–25 268)
			Plasma	61.7 (55.0–68.3)	26.5 (23.1–30.0)	20.5 (17.7–23.3)	72.1 (61.6–82.5)	31.1 (25.8–36.5)	24.1 (19.8–28.4)

In comparing the different skin conditions, the accumulation of CP in SC increases as the skin condition improves from lesional to non‐lesional to normal skin. Additionally, the systemic concentrations decreased as the skin condition improves. On average, CP plasma concentrations were 3.7‐fold higher when applied to lesional skin compared to normal skin.

Based on a dose of 1.2 mg/cm^2^, the weekly dose of CP was found to range from 7.7 g to 91 g weekly for an application area of 5% to 60%, respectively. Therefore, if CP were applied to an adult over 40% of the BSA, then this would exceed the maximum recommended weekly dose of CP (i.e., 50 g weekly).

## 4 DISCUSSION

Clobetasol propionate exerts anti‐inflammatory, immunosuppressive and antimitotic effects influencing the growth, differentiation and function of various cells and inhibiting cytokine production,^40^ all of which are relevant for symptomatic relief of conditions such as AD. Yet, little is known about the pharmacokinetic properties of CP at the dermal level, where its primary pharmacological activity occurs. In this study, we have used a PBPK modelling and simulation approach to characterize the pharmacokinetics of CP, considering the extent of skin involvement as well as the site and duration of treatment. Despite the limited availability of clinical data in healthy subjects and AD patients, our PBPK model provides valuable insights into the complex processes that determine the efficacy and safety profile of CP. Most importantly, it can be used to predict local and systemic exposures to CP under various clinical scenarios associated with the use of topical dosage forms, which would be challenging or impractical to assess through traditional clinical studies.

From a technical perspective, we have shown through standard statistical procedures that the model accurately predicts both local and systemic exposure following IV and topical application of the cream and ointment formulations. Both local (SC) and systemic (plasma) concentrations were used to verify the PBPK model, providing confidence in the predictions of CP at both sites. Recently, a PBPK model was developed using CP concentrations in the dermis.^24^ This PBPK model of CP was developed using the Transdermal Compartmental Absorption and Transit (TCAT) model within GastroPlus,^24^ where the SC and dermis were divided into 20 sub‐layers. Utilizing pharmacokinetic data in the dermis of psoriasis patients,^41^ the CP concentrations in the dermis were found to match the observed data at dermis sub‐layers 15–17. As the single‐layer dermis model was used in the present study, it was not possible to predict the concentration of CP at any given sub‐layer of the dermis.

From a clinical pharmacology perspective, we have found that the plasma half‐life of CP in healthy subjects is four times longer following topical use (20.8 h) compared to an IV dose (5.2 h). This indicates the presence of flip‐flop (zero‐order) kinetics, where the slow and continuous absorption of CP through the skin is much slower than the rate of elimination. This finding has potential clinical implications, as the use of CP twice a day may not be necessary. Previous studies have assumed that CP had a slow rate of elimination as cortisol levels remain suppressed 96 h after the dose.^7^, ^42^ The previous PBPK model of CP, did not include pharmacokinetic data following systemic administration, which would separate the effects of dermal absorption from the distribution and elimination of the drug.^24^ By contrast, with data following IV dosing and topical application, we now know that the systemic clearance of CP is 27.6 L/h and that the slow elimination of the drug is due to the continuous absorption of CP through the skin.

The development of a skin barrier impairment model provided the opportunity to predict CP exposure in the target population. The use of different formulations (cream *vs*. ointment) and occlusion had a minimal effect on the CP concentration‐time profiles in plasma. As expected, the bioavailability of CP varies with lesional, non‐lesional and normal skin. For lesional skin, as the skin barrier function is impaired, more of the drug passes through the skin which increases the systemic concentrations of CP. On the other hand, the CP concentrations in the skin are higher for normal skin compared to lesional skin, as the skin acts as a more effective barrier. The SC in subjects with normal skin can retain more of the drug, resulting in lower CP concentrations in plasma. It is worth noting that the PBPK predictions in the adult AD population were evaluated using three separate studies. The model underpredicted the systemic concentrations for the two patients with 80% and 95% of BSA affected by AD.^7^ In a separate study,^8^ overprediction of the systemic concentrations occurred for one patient (Patient B). However, this discrepancy is likely due to issues with compliance to the prescribed dose, as the concentrations of CP were much lower compared to Patient A, despite receiving higher doses of CP.

Interestingly, the application area was found to have a big impact on the pharmacokinetics of CP. An increase in application area increased both local and systemic CP concentrations for lesional, non‐lesional and normal skin. We have found the daily application of CP over an area larger than 30% of the BSA will exceed the maximum recommended dose of 50 g/week. Although a treatment duration of 2 weeks was simulated without varying the application surface area, the lesional skin is expected to reduce as the patients respond to treatment. This would lead to a reduced dose of CP cream or ointment, thereby reducing systemic exposure.

## 5 LIMITATIONS

The formulation attributes of CP were not readily available, and this lack of information required certain assumptions in the model development process. In particular, the Kp_disp:cont_ parameter was based on the solubility of the drug in the different phases. The uncertainty in the value of this parameter was also reported in a published PBPK model of CP, which explored of a range of values of Kp_disp:cont_ (3.65–32 based on K_disp,w_ of 357–3162).^24^ However, as the PBPK model utilized a different absorption model in a different software platform (Transdermal Compartmental Absorption & Transit (TCAT) Model, GastroPlus), there are likely to be differences in the parameter values due to differences in the parameterization of the PBPK models.

There are in vitro permeation test (IVPT) datasets available in the literature for CP.^27^, ^43^ However, the IVPT module in Simcyp, which enables parameter optimization by fitting model parameters to IVPT receptor profiles, was not available and is a limitation of the study. Furthermore, the dermal parameters were only optimized using CP concentration data in SC and in plasma.

The studies used to develop and verify the PBPK model had a limited sample size (<10 subjects), and large doses were used (>20 g daily), which exceeds the recommended maximum dose of CP. Additionally, the studies used to verify the model only specified the total dose of CP cream or ointment and not the area of application or the site of application. Therefore, assumptions were made on the application area, which was estimated using the rule of nines.^20^ However, as the model was verified using different clinical studies in healthy subjects and patients with AD, this provides confidence in the pharmacokinetic predictions in these population groups.

While the duration of application of CP was known for some studies (Study B (part 1 and 2) and Study D (part 2)),^10^, ^19^ this was assumed to be 24 h for studies that did not report it. That is, it was assumed that CP remained in contact with the skin for 24 h, although the more likely clinical scenario is that the formulation is subject to rubbing or being washed off throughout the day.

The development of the skin impairment was based on available information; however, it is a simplification of the various other physiological changes (e.g., altered skin blood flow) that occurs with AD. For the two patients with severe AD (80% and 95% of the BSA),^7^ the underprediction may be due to not accounting for other physiological changes with more severe disease. On the other hand, as a large proportion of the BSA is affected by the disease, it is also possible that CP was applied to sensitive body sites associated with higher dermal absorption (e.g., face and neck), which was not considered in the model.

## 6 CONCLUSIONS

CP is a highly effective treatment for skin disorders, particularly for skin disorders that do not respond to less potent TCS. Our work complements current guidelines based on a maximum recommended dose per week^1^, ^2^ and FTU^35^ by providing an indication of local and systemic exposures to CP with increasing application area. Our study suggests that CP should not be applied to an area of more than 30% of the BSA per day as this would exceed the maximum recommended dose (50 g/week) for 2–4 weeks. Whilst the current findings may benefit from further confirmatory evidence of CP exposure in patients with AD receiving therapeutic doses of CP, this study illustrates the importance of modelling and simulation for the characterization of the pharmacokinetics of topical drugs in dermatology.

## AUTHOR CONTRIBUTIONS

JD was involved in the analysis, interpretation of study data, drafting and review of the manuscript. SD, GO and AM were involved in study concept, the interpretation of study data, and critical revision of the manuscript. AC and EB were involved in interpretation of the study results, clinical implications and critical revision of the manuscript. ODP was involved in the study concept/design and interpretation of study data, drafting and critical revision of the manuscript. All authors had access to the study data, take responsibility for the accuracy of the analysis, contributed to data interpretation, reviewed, and contributed to the content of the manuscript, and had authority in the decision to submit the manuscript.

## CONFLICT OF INTEREST STATEMENT

JD, SD, GO, AM and ODP are GSK employees and hold financial equities in GSK. AC and EB received personal payment from GSK for external expert consultations to adapt modelling procedures and for interpreting results.

## Supporting information


**Table S1** Oral PBPK parameters for clobetasol propionate.
**Figure S1** Observed and predicted  concentration–time profile in plasma following a single oral dose of 2 mg CP to six male subjects.
**Table S2** Observed and predicted secondary pharmacokinetic parameters following a single oral dose of 2 mg CP.
**Figure S2** The structure of the MPML MechDermA model.
**Table S3** The impact of each individual parameter on the AUC in plasma.
**Table S4** Trial design characteristics for CP cream (0.05%) in the healthy adult population using Simcyp V22.
**Table S5** Trial design characteristics for CP ointment (0.05%) in the healthy adult population using Simcyp V22.
**Table S6** Trial design characteristics for CP ointment (0.05%) in the adult population with AD using Simcyp V22.
**Table S7** Predicted estimates of the apparent plasma half‐life (*t*_1/2_).

## Data Availability

Data sharing is not applicable to this article as no new data were created or analysed in this study.

## References

[1] GlaxoSmithKline . 2024. Dermovate cream SmPC. Available from: https://www.medicines.org.uk/emc/product/939/smpc. Accessed December 10, 2024.

[2] GlaxoSmithKline . 2024. Dermovate ointment SmPC. Available from: https://www.medicines.org.uk/emc/product/940/smpc. Accessed 10 December 10, 2024.

[3] Prete A , Bancos I . Glucocorticoid induced adrenal insufficiency. BMJ. 2021;374:n1380. doi:10.1136/bmj.n1380 34253540

[4] Carruthers JA , August PJ , Staughton RC . Observations on the systemic effect of topical clobetasol propionate (Dermovate). Br Med J. 1975;4(5990):203‐204. doi:10.1136/bmj.4.5990.203 1191997 PMC1674965

[5] Wood Heickman LK , Davallow Ghajar L , Conaway M , Rogol AD . Evaluation of hypothalamic‐pituitary‐adrenal axis suppression following cutaneous use of topical corticosteroids in children: a meta‐analysis. Horm Res Paediatr. 2018;89(6):389‐396. doi:10.1159/000489125 29898449

[6] Levin C , Maibach HI . Topical corticosteroid‐induced adrenocortical insufficiency: clinical implications. Am J Clin Dermatol. 2002;3(3):141‐147. 11978135 10.2165/00128071-200203030-00001

[7] Hehir M , Du Vivier A , Eilon L , Danie MJ , Shenoy EV . Investigation of the pharmacokinetics of clobetasol propionate and clobetasone butyrate after a single application of ointment. Clin Exp Dermatol. 1983;8(2):143‐151. 6851236 10.1111/j.1365-2230.1983.tb01758.x

[8] Sparidans RW , van Velsen SG , de Roos MP , Schellens JH , Bruijnzeel‐Koomen CA , Beijnen JH . Liquid chromatography‐tandem mass spectrometric assay for clobetasol propionate in human serum from patients with atopic dermatitis. J Chromatogr B. 2010;878(23):2150‐2154. 10.1016/j.jchromb.2010.06.011 20605116

[9] van Velsen SG , De Roos MP , Haeck IM , Sparidans RW , Bruijnzeel‐Koomen CA . The potency of clobetasol propionate: serum levels of clobetasol propionate and adrenal function during therapy with 0.05% clobetasol propionate in patients with severe atopic dermatitis. J Dermatolog Treat. 2012;23(1):16‐20. doi:10.3109/09546634.2010.534127 21254880

[10] Harding SM , Sohail S , Busse MJ . Percutaneous absorption of clobetasol propionate from novel ointment and cream formulations. Clin Exp Dermatol. 1985;10(1):13‐21. doi:10.1111/j.1365-2230.1985.tb02546.x 3987081

[11] Jamei M , Dickinson GL , Rostami‐Hodjegan A . A framework for assessing inter‐individual variability in pharmacokinetics using virtual human populations and integrating general knowledge of physical chemistry, biology, anatomy, physiology and genetics: a tale of “bottom‐up” vs “top‐down” recognition of covariates. Drug Metab Pharmacokinet. 2009;24(1):53‐75. doi:10.2133/dmpk.24.53 19252336

[12] Patel N , Clarke JF , Salem F , et al. Multi‐phase multi‐layer mechanistic dermal absorption (MPML MechDermA) model to predict local and systemic exposure of drug products applied on skin. CPT Pharmacometrics Syst Pharmacol. 2022;11(8):1060‐1084. doi:10.1002/psp4.12814 35670226 PMC9381913

[13] Wisniowska B , Linke S , Polak S , Bielecka Z , Luch A , Pirow R . Physiologically based modelling of dermal absorption and kinetics of consumer‐relevant chemicals: a case study with exposure to bisphenol A from thermal paper. Toxicol Appl Pharmacol. 2023;459:116357. doi:10.1016/j.taap.2022.116357 36572228

[14] Tsakalozou E , Babiskin A , Zhao L . Physiologically‐based pharmacokinetic modeling to support bioequivalence and approval of generic products: a case for diclofenac sodium topical gel, 1. CPT Pharmacometrics Syst Pharmacol. 2021;10(5):399‐411. doi:10.1002/psp4.12600 33547863 PMC8129718

[15] Glaxo Group Research Limited . The bioavailability and pharmacokinetics of clobetasol propionate after 2 mg oral and intravenous doses (Report No. GMH/83/006). Greenford, 1983.

[16] Glaxo Group Resarch Limited . A comparison of cream formulations containing clobetasol propionate in butyl adipate and in propylene glycol for effect on plasma levels of clobetasol propionate and cortisol (Report No. GMH/82/017). Greenford, 1982.

[17] Glaxo Group Research Limited . Plasma levels of fluticasone propionate, betamethasone and clobetasol propionate following topical application of cream and ointment preparations (Report No. GMH/90/015). Greenford, 1990.

[18] Glaxo Group Resarch Limited . Human volunteer trial to investigate the plasma levels of clobetasol 17‐propionate after single application of Dermovate cream and ointment with and without the use of occlusion (Report No. HVT/79/16). Greenford, 1979.

[19] Au WL , Skinner M , Kanfer I . Comparison of tape stripping with the human skin blanching assay for the bioequivalence assessment of topical clobetasol propionate formulations. J Pharm Pharm Sci. 2010;13(1):11‐20. 20456826 10.18433/j3c01r

[20] Yasti AC , Senel E , Saydam M , Ozok G , Coruh A , Yorganci K . Guideline and treatment algorithm for burn injuries. Ulus Travma Acil Cerrahi Derg. 2015;21(2):79‐89. doi:10.5505/tjtes.2015.88261 25904267

[21] Rohatgi, A. 2022. WebPlotDigitizer v4.6, California, USA.

[22] Jones H , Rowland‐Yeo K . Basic concepts in physiologically based pharmacokinetic modeling in drug discovery and development. CPT Pharmacometrics Syst Pharmacol. 2013;2(8):e63. doi:10.1038/psp.2013.41 23945604 PMC3828005

[23] Rodgers T , Rowland M . Physiologically based pharmacokinetic modelling 2: predicting the tissue distribution of acids, very weak bases, neutrals and zwitterions. J Pharm Sci. 2006;95(6):1238‐1257. doi:10.1002/jps.20502 16639716

[24] van Osdol WW , Novakovic J , Le Merdy M , et al. Predicting human dermal drug concentrations using PBPK modeling and simulation: clobetasol propionate case study. AAPS PharmSciTech. 2024;25(3):39. doi:10.1208/s12249-024-02740-x 38366149

[25] Fauzee AFB . Development, manufacture, and assessment of clobetasol 17‐propionate cream formulationsMaster's thesis. Rhodes University; 2011.

[26] Cheruvu HS , Liu X , Grice JE , Roberts MS . Modeling percutaneous absorption for successful drug discovery and development. Expert Opin Drug Discovery. 2020;15(10):1181‐1198. 10.1080/17460441.2020.1781085 32584615

[27] Soares KCC , de Souza WC , de STL , da Cunha‐Filho MSS , Gelfuso GM , Gratieri T . Comparison of clobetasol propionate generics using simplified in vitro bioequivalence method for topical drug products. Curr Drug Deliv. 2018;15(7):998‐1008. 29165079 10.2174/1567201814666171120125333

[28] Yamamoto Y , Onuki Y , Fukami T , Koide T . Comparison of various pharmaceutical properties of clobetasol propionate cream formulations—considering stability of mixture with moisturizer. J Pharm Health Care Sci. 2020;6(1):1. doi:10.1186/s40780-020-0158-y 32015896 PMC6990562

[29] Johnson ME , Blankschtein D , Langer R . Evaluation of solute permeation through the stratum corneum: lateral bilayer diffusion as the primary transport mechanism. J Pharm Sci. 1997;86(10):1162‐1172. doi:10.1021/js960198e 9344175

[30] Seidenari S , Giusti G . Objective assessment of the skin of children affected by atopic dermatitis: a study of pH, capacitance and TEWL in eczematous and clinically uninvolved skin. Acta Derm Venereol. 1995;75(6):429‐433. doi:10.2340/0001555575429433 8651017

[31] Polanska A , Danczak‐Pazdrowska A , Silny W , Jenerowicz D , Olek‐Hrab K , Osmola‐Mankowska A . Nonlesional skin in atopic dermatitis is seemingly healthy skin: observations using noninvasive methods. Wideochir Inne Tech Maloinwazyjne. 2013;8(3):192‐199. 24130632 10.5114/wiitm.2011.33633 PMC3796717

[32] Janssens M , van Smeden J , Puppels GJ , Lavrijsen AP , Caspers PJ , Bouwstra JA . Lipid to protein ratio plays an important role in the skin barrier function in patients with atopic eczema. Br J Dermatol. 2014;170(6):1248‐1255. doi:10.1111/bjd.12908 24641443

[33] Werner Y , Lindberg M . Transepidermal water loss in dry and clinically normal skin in patients with atopic dermatitis. Acta Derm Venereol. 1985;65(2):102‐105. doi:10.2340/0001555565102105 2408409

[34] Kashibuchi N , Hirai Y , O'Goshi K , Tagami H . Three‐dimensional analyses of individual corneocytes with atomic force microscope: morphological changes related to age, location and to the pathologic skin conditions. Skin Res Technol. 2002;8(4):203‐211. doi:10.1034/j.1600-0846.2002.00348.x 12423538

[35] Aung T , Aung ST . Selection of an effective topical corticosteroid. Aust J Gen Pract. 2021;50(9):651‐655. doi:10.31128/AJGP-07-20-5507 34462770

[36] Hansen S , Lehr CM , Schaefer UF . Improved input parameters for diffusion models of skin absorption. Adv Drug Deliv Rev. 2013;65(2):251‐264. doi:10.1016/j.addr.2012.04.011 22626979

[37] Shatkin JA , Brown HS . Pharmacokinetics of the dermal route of exposure to volatile organic chemicals in water: a computer simulation model. Environ Res. 1991;56(1):90‐108. doi:10.1016/S0013-9351(05)80112-4 1915193

[38] Chen L , Han L , Saib O , Lian G . In silico prediction of percutaneous absorption and disposition kinetics of chemicals. Pharm Res. 2015;32(5):1779‐1793. doi:10.1007/s11095-014-1575-0 25407547

[39] Polak S , Patel N . Combining machine learning and mechanistic modeling approaches to solve real life problems—assessment of the local tissue binding and its influence on the systemic exposure after topical application of drugs. In: ACoP9, October 6–10, 2018, San Diego, USA, 2018.

[40] Pels R , Sterry W , Lademann J . Clobetasol propionate—where, when, why? Drugs Today (Barc). 2008;44(7):547‐557. doi:10.1358/dot.2008.44.7.1122221 18806904

[41] Bodenlenz M , Dragatin C , Liebenberger L , et al. Kinetics of clobetasol‐17‐propionate in psoriatic lesional and non‐lesional skin assessed by dermal open flow microperfusion with time and space resolution. Pharm Res. 2016;33(9):2229‐2238. doi:10.1007/s11095-016-1960-y 27271272 PMC4967091

[42] Staughton RC , August PJ . Cushing's syndrome and pituitary‐adrenal suppression due to clobetasol propionate. Br Med J. 1975;2(5968):419‐421. doi:10.1136/bmj.2.5968.419 1125562 PMC1681806

[43] Lehman PA , Franz TJ . Assessing topical bioavailability and bioequivalence: a comparison of the in vitro permeation test and the vasoconstrictor assay. Pharm Res. 2014;31(12):3529‐3537. doi:10.1007/s11095-014-1439-7 25005736

