# Supplementary material for: Characterizing local and systemic exposure to clobetasol propionate in healthy subjects and patients with atopic dermatitis
Source: Br J Clin Pharmacol. 2025 Jul 11;91(11):3150–66. doi: 10.1002/bcp.70102 (PMC12569561; doi:10.1002/bcp.70102)
Supplement: Supplementary file 1 — Table S1 Oral PBPK parameters for clobetasol propionate. Figure S1 Observed and predicted concentration–time profile in plasma following a single oral dose of 2 mg CP to six male subjects. Table S2 Observed and predicted secondary pharmacokinetic parameters following a single oral dose of 2 mg CP. Figure S2 The structure of the MPML MechDermA model. Table S3 The impact of each individual parameter on the AUC in plasma. Table S4 Trial design characteristics for CP cream (0.05%) in the healthy adult population using Simcyp V22. Table S5 Trial design characteristics for CP ointment (0.05%) in the healthy adult population using Simcyp V22. Table S6 Trial design characteristics for CP ointment (0.05%) in the adult population with AD using Simcyp V22. Table S7 Predicted estimates of the apparent plasma half‐life (t 1/2). [file BCP-91-3150-s001.pdf]

Supplementary Information

Characterising local and systemic exposure to clobetasol propionate in healthy subjects and patients with atopic dermatitis

Janna K Duong[a], Sven van Dijkman[b], Gary Ong[c], Alexandra Marta[c], Adriana Ceci[d], Ernesto Bonifazi[e], Oscar Della Pasqua[f]

[a]Clinical Pharmacology Modelling & Simulation, GSK, Australia

[b]Clinical Pharmacology Modelling & Simulation, GSK, UK

[c]Pharma Research and Development, Global Medical, GSK, Singapore

[d]Pharmacological Research Foundation 'Gianni Benzi', Italy

[e]Past professor of Pediatric Dermatology "Aldo Moro" University Bari Italy

[f]Clinical Pharmacology & Therapeutics Group, University College London, London, UK.

Development of Oral PBPK Model for clobetasol propionate

A first-order (FO) rate of absorption model was applied to the PBPK model of clobetasol to predict clobetasol exposure following a single oral dose of 2 mg. The parameter values used to develop the oral model are shown in Supplementary Table S1.

The goodness-of-fit plot is depicted in Figure S1, whereas the observed and predicted pharmacokinetic parameters are shown in Table S2. There was good agreement between the observed and predicted concentrations of clobetasol following a single oral dose and the predictions were within the acceptance criteria.

Supplementary Table S1 Oral PBPK parameters for clobetasol propionate

Absorption Parameter	Value	Assumptions
f_a	0.5 (30% CV)	Based on mean oral availability data from Study A (50%)
k_a (1/h)	0.86 (30% CV)	Using parameter estimation tool in Simcyp based on oral data (Study A)
Lag time (h)	0 (30% CV)	There is no information on lag time of CP following oral administration. As there is limited data on patients receiving oral dose of CP (n = 6, Study A), a lag time of 0 h was assumed to simplify the model.
$f_{u,gut}$	0.0026	Simcyp QSAR Predicted
Q_{gut} (L/h)	15.015	Simcyp QSAR Predicted
Peff,man (10-4 cm/s)	4.804	Simcyp QSAR Predicted

F_a, fraction available from dosage form; k_a, absorption rate constant; $f_{u,gut}$, unbound fraction of drugs in enterocytes; Q_{gut}, nominal flow in gut model.

Supplementary Figure S1 Observed and predicted concentration-time profile in plasma following a single oral dose of 2 mg CP to 6 male subjects

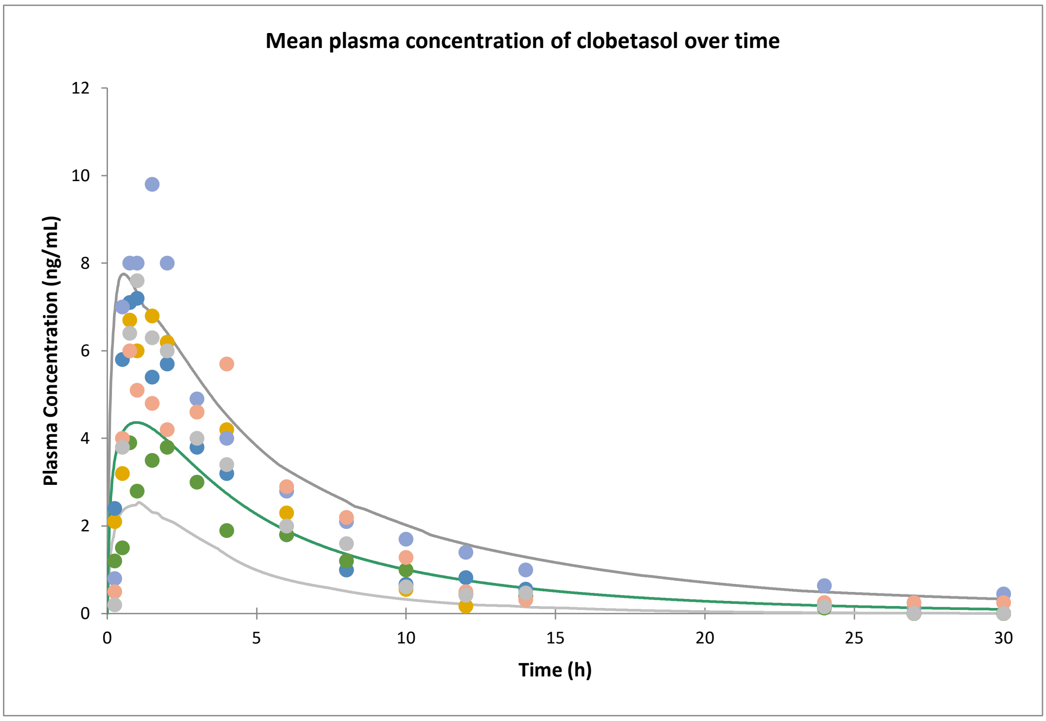

Green line represents the median of the simulated clobetasol concentrations, grey lines represent the 5th and 95th percentiles of simulated clobetasol concentrations, the dots represent the observed concentrations.

Supplementary Table S2 Observed and predicted secondary pharmacokinetic parameters following a single oral dose of 2 mg CP

Formulation	Parameter	Observed Mean (SE)	Predicted Mean (95% CI)	Ratio (Pred/Obs)
Oral	**Tmax (h)[a]**	1.1 (0.1)	1.1 (0.4, 3.2)	1.00
	**Cmax (ng/mL)**	6.9 (0.8)	4.5 (3.9 – 4.6)	0.65
	**t½ (h)**	4.1 (0.1)	5.7 (4.8 – 5.9)	1.39
	**$AUC_{0\text{-}30h}$ (ng.h/mL)**	36.3 (2.7)	32.0 (26.3 – 32.7)	0.88
	**$AUC_{0\text{-}INF}$ (ng.h/mL)**	37.6 (3.5)	33.0 (26.9 – 33.8)	0.88

$AUC_{0\text{-}30h}$ area under the plasma concentration-time curve from time zero to 30 hours, $AUC_{0\text{-}INF}$ area under the plasma concentration-time curve from time zero extrapolated to infinity, *t½* apparent half-life of elimination, *tmax* time to peak concentration.

a. Tmax expressed as median (range)

Supplementary Figure S2 The structure of the MPML MechDermA model

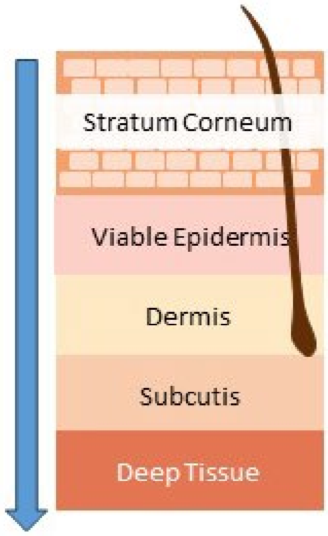

The MPML MechDermA model accounts for one-dimensional partitioning and diffusion processes. The MPML MechDermA model is based on eight components: 1) formulation; (2) stratum corneum; (3) viable epidermis; (4) dermis; (5) hair follicle; (6) subcutis; (7) muscle (deep tissue) and (8) local vasculature (blood circulation). The stratum corneum (SC) is modeled as a "brick-and-mortar" structure, which represents the corneocytes embedded within the intercellular lipid matrix. The viable epidermis, dermis, subcutis and muscle are modelled as a single well-stirred compartment.

The hair follicle extend from the surface of the skin to the dermis. The hair follicle acts as a shunt that take the drug from the surface of the skin and exchanges it with the dermis. Details of the MPML MechDermA model was obtained from [Patel, 2022].

Supplementary Table S3 The impact of each individual parameter on the AUC in plasma

Parameter	Mean CP AUC in plasma (ng.h/mL) (5th-95th percentiles)	
	No modification (normal skin)	Parameter modification for the skin impairment model (lesional skin)
Skin Thickness	3.3 (3.0 – 3.6)	7.7 (7.0 – 8.4)
Hydration	3.3 (3.0 – 3.6)	3.4 (3.1 – 3.7)
pH	3.3 (3.0 – 3.6)	3.3 (3.0 – 3.6)
Corneocyte	3.3 (3.0 – 3.6)	3.8 (3.5 – 4.1)

Supplementary Table S4 Trial design characteristics for CP cream (0.05%) in the healthy adult population using Simcyp V22

	[Au, 2010]	Study B (part 1)[a]	Study B (part 2)[a]	Study C[b]	Study D (part 1)[c]
**Modelling Step**	Model Development	Model Development	Model Verification	Model Verification	Model Verification
**Dosing Regimen**	5.5 mg/cm^2	30 g two application	15 g once daily	12.5 g twice daily application	25 g single application
**Duration of Application**	2 h	13 h		-	13 h
**Use of Occlusion**	No	No		No	Study arm 1: No<br>Study arm 2: Yes
**Mean BSA (cm^2)**	N/A	19300		19300	19000
**Total Area of Application (cm^2) (% of BSA)**	4	11580 (60%)		9650 (50%)	11400 (60%)
**Application Sites and Application Area[d]**	Forearm inner: 4 cm^2	Abdomen: 2509 cm^2<br>Back: 2509 cm^2<br>Leg (upper/thigh): 3474 cm^2<br>Leg (lower): 3088 cm^2		Abdomen: 2509 cm^2<br>Back: 2509 cm^2<br>Upper arm: 1544 cm^2<br>Forearm inner: 579 cm^2<br>Forearm outer: 579 cm^2	Abdomen: 2470 cm^2<br>Back: 2470 cm^2<br>Leg (upper/thigh): 3420 cm^2<br>Leg (lower): 3040 cm^2
**Total Formulation Volume (mL)[e]**	0.02	27.27	13.64	11.36	22.73
**Formulation Volume per Site (mL)**	0.02	Abdomen: 6<br>Back: 6<br>Leg (upper/thigh): 8.18<br>Leg (lower): 7.09	Abdomen: 3<br>Back: 3<br>Leg (upper/thigh): 4.09<br>Leg (lower): 3.55	Abdomen: 3.69<br>Back: 3.69<br>Upper arm: 2.28<br>Forearm Inner: 0.85<br>Forearm Outer: 0.85	Abdomen: 4.55<br>Back: 4.55<br>Leg (upper/thigh): 6.82<br>Leg (lower): 6.82
**Thickness of formulation (cm)**	0.005	0.002	0.001	0.001	0.002
**Age range (y)**	20 – 36	22 – 41		19 - 48	20 – 65
**Sex**	% Female = 50	% Female = 0		% Female = 0	% Female = 0
**Subject numbers in clinical study**	30	7		6	6
**Number of simulation trials**	4	20		20	20
**Population size**	120	140		120	120

a. Whole body application excluding face, arms, and genital area. Assume neck, hands and feet excluded. Total area of application is 60% of mean BSA (1.93 m^2) based on the rule of nines [Yasti, 2015]. CP distribution based on affected sites: back (22%), abdomen (22%), leg upper (30%), and leg lower (26%).

b. Dermovate cream applied evenly to the torso and arms. Assume 50% of mean BSA (1.93 m^2) for area of application. Total area = 0.965 m^2. Body site distribution of affected areas: back (32.5%), abdomen (32.5%), upper arm (20%), forearm inner (7.5%) and forearm outer (7.5%).

c. Whole body application excluding face, neck, feet, both arms and genital area. Assume hands excluded. Assume 60% of mean BSA (1.90m^2) for area of application based on the rule of nines [Yasti, 2015]. Total BSA affected is 1.14 m^2. Body site distribution of affected areas: back (20%), abdomen (20%), leg upper (30%), leg lower (30%).

d. The abdomen site in Simcyp was used to represent the entire front torso (chest and abdomen).

e. Formulation volume (mL) = Amount of drug (g) / formulation density (g/mL).

Supplementary Table S5 Trial design characteristics for CP ointment (0.05%) in the healthy adult population using Simcyp V22

Study	Study D (part 2)[a]	[Harding, 1985][b]
Modelling Step	Model Development	Model Verification
Dosing Regimen	25 g single application	30 g two applications
Duration of Application	13	13
Use of Occlusion	No	No
Mean BSA (cm^2)	19000	19000
Total Area of Application (cm^2) (% of BSA)	11400 (60%)	12920 (68%)
Application Sites (cm^2)[c]	Abdomen: 2470<br>Back: 2470<br>Leg (upper/thigh): 3420 cm^2<br>Leg (lower): 3040 cm^2	Abdomen: 2470<br>Back: 2470<br>Leg (upper/thigh): 3420<br>Leg (lower): 3040<br>Upper arm: 1520
Formulation Density (g/mL)	0.8	0.8
Total Formulation Volume (mL)[f]	31.25	37.5
Formulation Volume per Site (mL)[d]	Abdomen: 6.25<br>Back: 6.25<br>Leg (upper/thigh): 9.38<br>Leg (lower): 9.38	Abdomen: 7.5<br>Back: 7.5<br>Leg (upper/thigh): 9<br>Leg (lower): 9<br>Upper arm: 4.5
Thickness of formulation (cm)	0.003	0.003
Population	Sim-Healthy Volunteer	Sim-Healthy Volunteer
Age range (y)	20 – 65	23 - 46
Sex	% Female = 0	% Female = 0
Subject numbers in clinical study	6	8
Number of simulation trials	20	15
Population size	120	120

a. Whole body application excluding face, neck, feet, both arms and genital area. Assume 60% of mean BSA (1.90m^2) for area of application based on the rule of nines [Yasti, 2015]. Total BSA affected is 1.14 m^2. Body site distribution of affected areas: back (20%), abdomen (20%), leg upper (30%), leg lower (30%).
b. Whole body application excluding face, neck, feet, and genital area. Assume 68% of mean BSA (1.90m^2) for area of application based on the rule of nines [Yasti, 2015]. Total BSA affected is 1.40 m^2. Body site distribution of affected areas: back (20%), abdomen (20%), leg upper (24%), leg lower (24%), upper arm (12%).
c. The abdomen site in Simcyp was used to represent the entire front torso (chest and abdomen).
d. Formulation volume (ml) = Amount of drug applied (g) / formulation density (g/mL).

Supplementary Table S6 Trial design characteristics for CP ointment (0.05%) in the adult population with AD using Simcyp V22

Study	[Hehir, 1983]	[Sparidans, 2010]	[van Velsen, 2012]
Modelling Step	Model Verification	Model Verification	Model Verification
Dosing Regimen	25 g single application	15 g daily	Group A: 25 g single application<br>Group B: 25 g two applications
Duration of Application	24	24	24
Use of Occlusion	No	No	No
Mean BSA (cm^2)	19000	17000	18000
Total Area of Application (cm^2) (% of BSA)	11400 (60%)	12580 (74%)	13320 (74%)
Application Sites (cm^2)[c]	Abdomen: 2470<br>Back: 2470<br>Leg (upper/thigh): 3420 cm^2<br>Leg (lower): 3040 cm^2	Abdomen: 2210<br>Back: 2210<br>Leg (upper/thigh): 3060<br>Leg (lower): 2720<br>Upper arm: 1360<br>Forearm Inner/Outer: 510	Abdomen: 2340<br>Back: 2340<br>Leg (upper/thigh): 3240<br>Leg (lower): 2880<br>Upper arm: 1440<br>Forearm Inner/Outer: 540
Formulation Density (g/mL)	0.8	0.8	0.8
Total Formulation Volume (mL)[f]	31.25	18.75	31.25
Formulation Volume per Site (mL)[d]	Abdomen: 6.25<br>Back: 6.25<br>Leg (upper/thigh): 9.38<br>Leg (lower): 9.38	Abdomen: 3.29<br>Back: 3.29<br>Leg (upper/thigh): 4.56<br>Leg (lower): 4.05<br>Upper arm: 2.03<br>Forearm Inner/Outer: 0.76	Abdomen: 5.49<br>Back: 5.49<br>Leg (upper/thigh): 7.6<br>Leg (lower): 6.76<br>Upper arm: 3.38<br>Forearm Inner/Outer: 1.27
Thickness of formulation (cm)	0.003	0.003	0.002
Population	Sim-Healthy Volunteer	Sim-Healthy Volunteer	Sim-Healthy Volunteer
Age range (y)	20 – 65	Patient A: 19<br>Patient B: 66	18 - 71
Sex	% Female = 0	% Female = 100	% Female = 60%
Subject numbers in clinical study	6	2	16
Number of simulation trials	20	50	10
Population size	120	100	160

a. Whole body application excluding face, neck, feet, both arms and genital area. Assume 60% of mean BSA (1.90m^2) for area of application based on the rule of nines [Yasti, 2015]. Total BSA affected is 1.14 m^2. Body site distribution of affected areas: back (20%), abdomen (20%), leg upper (30%), leg lower (30%).
b. Whole body application excluding face, neck, feet, and genital area. Assume 60% of mean BSA (1.90m^2) for area of application based on the rule of nines [Yasti, 2015]. Total BSA affected is 1.40 m^2. Body site distribution of affected areas: back (20%), abdomen (20%), leg upper (24%), leg lower (24%), upper arm (12%).
c. The abdomen site in Simcyp was used to represent the entire front torso (chest and abdomen).
d. Formulation volume (ml) = Amount of drug applied (g) / formulation density (g/mL).

Supplementary Table S7: Predicted estimates of the apparent plasma half-life (t½)

Study	Formulation	Dose (g)	Dosing Regimen	Parameter	Predicted Mean (95% CI)
Study A	IV	0.02	Single dose	t½ (h)	5.2 (4.9 – 5.6)
Study B (part 1)	Cream	30	Two applications	t½ (h)	12.0 (7.7 – 26.3)
Study B (part 2)	Cream	15	Once daily, multiple dose	t½ (h)	52.5 (36.3 – 68.6)
Study C	Cream	12.5	Twice daily, multiple dose	t½ (h)	11.5 (7.2 – 15.7)
Study D (part 1)	Cream	25	Single application	t½ (h)	15.8 (13.6 – 17.9)
Study D (part 2)	Ointment	25	Single application	t½ (h)	16.6 (12.5 – 20.7)
[Harding, 1985][a]	Ointment	30	Two applications	t½ (h)	16.1 (12.9 – 19.3)

The apparent t ½ was determined following the first dose of the drug over a dosage interval of 24 h. The large variability in the elimination t½ of CP is due to different amounts of drug applied to different body sites, which have different absorption profiles.

REFERENCES

Au WL, Skinner M, Kanfer I. Comparison of tape stripping with the human skin blanching assay for the bioequivalence assessment of topical clobetasol propionate formulations. *J Pharm Pharm Sci* 2010;13(1):11-20.

Harding SM, Sohail S, Busse MJ. Percutaneous absorption of clobetasol propionate from novel ointment and cream formulations. *Clin Exp Dermatol* 1985 Jan;10(1):13-21.

Hehir M, Du Vivier A, Eilon L, Danie MJ, Shenoy EV. Investigation of the pharmacokinetics of clobetasol propionate and clobetasone butyrate after a single application of ointment. *Clin Exp Dermatol* 1983 Mar;8(2):143-51.

Patel N, Clarke JF, Salem F, Abdulla T, Martins F, Arora S*, et al.* Multi-phase multi-layer mechanistic dermal absorption (MPML MechDermA) model to predict local and systemic exposure of drug products applied on skin. *CPT Pharmacometrics Syst Pharmacol* 2022 Aug;11(8):1060-84.

Sparidans RW, van Velsen SG, de Roos MP, Schellens JH, Bruijnzeel-Koomen CA, Beijnen JH. Liquid chromatography-tandem mass spectrometric assay for clobetasol propionate in human serum from patients with atopic dermatitis. *J Chromatogr B Analyt Technol Biomed Life Sci* 2010 Aug 1;878(23):2150-4.

van Velsen SG, De Roos MP, Haeck IM, Sparidans RW, Bruijnzeel-Koomen CA. The potency of clobetasol propionate: serum levels of clobetasol propionate and adrenal function during therapy with 0.05% clobetasol propionate in patients with severe atopic dermatitis. *J Dermatolog Treat* 2012 Feb;23(1):16-20.

Yasti AC, Senel E, Saydam M, Ozok G, Coruh A, Yorganci K. Guideline and treatment algorithm for burn injuries. *Ulus Travma Acil Cerrahi Derg* 2015 Mar;21(2):79-89.
